# New horizons in the diagnosis and management of Alzheimer’s Disease in older adults

**DOI:** 10.1093/ageing/afae005

**Published:** 2024-02-10

**Authors:** Helena Dolphin, Adam H Dyer, Laura Morrison, Susan D Shenkin, Tomas Welsh, Sean P Kennelly

**Affiliations:** Discipline of Medical Gerontology, School of Medicine, Trinity College Dublin, Dublin, Ireland; Institute of Memory and Cognition, Tallaght University Hospital, Dublin, Ireland; Discipline of Medical Gerontology, School of Medicine, Trinity College Dublin, Dublin, Ireland; Institute of Memory and Cognition, Tallaght University Hospital, Dublin, Ireland; Discipline of Medical Gerontology, School of Medicine, Trinity College Dublin, Dublin, Ireland; Institute of Memory and Cognition, Tallaght University Hospital, Dublin, Ireland; Ageing and Health Research Group, Advanced Care Research Centre, Usher Institute, University of Edinburgh, Edinburgh, UK; Bristol Medical School (THS), University of Bristol, Bristol, UK; RICE – The Research Institute for the Care of Older People, Bath, UK; Royal United Hospitals Bath NHS Foundation Trust, Bath, UK; Discipline of Medical Gerontology, School of Medicine, Trinity College Dublin, Dublin, Ireland; Institute of Memory and Cognition, Tallaght University Hospital, Dublin, Ireland

**Keywords:** Alzheimer’s disease, diagnosis, biomarkers, dementia, older people

## Abstract

Alzheimer’s Disease (ad) is the most common cause of dementia, and in addition to cognitive decline, it directly contributes to physical frailty, falls, incontinence, institutionalisation and polypharmacy in older adults. Increasing availability of clinically validated biomarkers including cerebrospinal fluid and positron emission tomography to assess both amyloid and tau pathology has led to a reconceptualisation of ad as a clinical–biological diagnosis, rather than one based purely on clinical phenotype. However, co-pathology is frequent in older adults which influence the accuracy of biomarker interpretation. Importantly, some older adults with positive amyloid or tau pathological biomarkers may never experience cognitive impairment or dementia. These strides towards achieving an accurate clinical–biological diagnosis are occurring alongside recent positive phase 3 trial results reporting statistically significant effects of anti-amyloid Disease-Modifying Therapies (DMTs) on disease severity in early ad. However, the real-world clinical benefit of these DMTs is not clear and concerns remain regarding how trial results will translate to real-world clinical populations, potential adverse effects (including amyloid-related imaging abnormalities), which can be severe and healthcare systems readiness to afford and deliver potential DMTs to appropriate populations. Here, we review recent advances in both clinical–biological diagnostic classification and future treatment in older adults living with ad. Advocating for access to both more accurate clinical–biological diagnosis and potential DMTs must be done so in a holistic and gerontologically attuned fashion, with geriatricians advocating for enhanced multi-component and multi-disciplinary care for all older adults with ad. This includes those across the ad severity spectrum including older adults potentially ineligible for emerging DMTs.

## Key Points

- Alzheimer’s Disease (ad) has traditionally been diagnosed based on clinical phenotype alone.
- Increasing availability of cerebrospinal fluid (CSF) and positron emission tomography (PET) biomarkers of amyloid and tau pathology has led to a reconceptualisation of ad as a clinical–biological diagnosis.
- In older adults, ‘pure’ad is the exception rather than the rule and biomarker interpretation may be more difficult.
- Novel Disease-Modifying Therapies (DMTs) targeting the fibrillar form of amyloid have demonstrated statistically significant effects in phase 3 trials in early ad, but the real-world impact of these effects in older adults with ad is unclear at present.
- In advocating for increased access to diagnostics and treatments, geriatricians need to advocate for all older adults with ad, especially those with more advanced stages of the disease who may be ineligible for potential new DMTs, they should become available.

## Introduction

Alzheimer’s Disease (ad) affects 5% of those aged 65 and up to one-third of those aged 90 years and older [1]. Not only does ad directly contribute to increased morbidity and mortality in older adults, it is also strongly associated with the development of several other geriatric syndromes including falls, delirium, urinary incontinence, polypharmacy and physical frailty [2–4]. For geriatricians, incorporating a Comprehensive Geriatric Assessment (CGA) approach to the assessment of both cognition and these frequently co-morbid syndromes is central to holistic care of older adults living with ad. Here, we explore evolving advances in the clinical–biological diagnosis and treatment of ad in older adults. While this discussion is restricted to ad in considering these recent developments, it should be remembered that ‘pure ad’ is the exception rather than the rule in older adults [5], and the presence of co-pathology contributing to both Mild Cognitive Impairment (MCI) and dementia is common in those presenting to a Memory Assessment Service (MAS) [6]. Given the global variability in structure of a MAS [7], the term MAS will refer to memory clinics and memory assessment and support services in hospital settings in this article.

### AD pathophysiology

**Figure 1:**
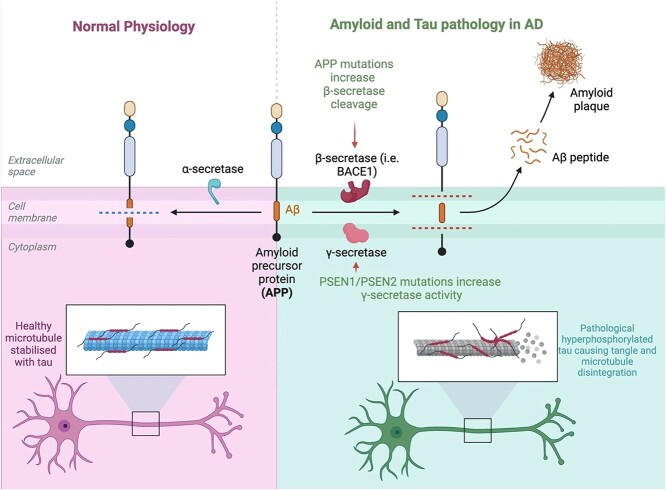
Amyloid and Tau processing under physiological conditions and in Alzheimer’s Disease. PSEN 1: Presenilin 1. PSEN 2: Presenilin 2. Aβ: Amyloid beta. BACE-1: Beta- site Amyloid precursor protein Cleaving Enzyme 1. Image adapted from “Cleavage of Amyloid Precursor Protein (APP)” by BioRender.com (2020).

The two most clearly defined pathological processes involved in ad involve amyloid plaque and hyper-phosphorylated tau accumulation. Amyloid Precursor Protein (APP) is cleaved by a membrane protein enzyme. In healthy states, it is cleaved by α-secretase and releases a non-toxic protein α-CTF [8]. When amyloid pathologically builds up, β- and γ- secretase cleave APP at various sites along the protein to release amyloid fibrils of various lengths. The most toxic of these is amyloid-β 42 (Aβ-42) which is released back into the extracellular space and causes a conformational change in other amyloid proteins that aggregate into oligomers, fibrils and ultimately plaques [9]. Tau is a microtubule-binding protein that stabilises axonal structures that shuttle nutrients and signals along the length of axons. Hyperphosphorylation of tau (p-tau) is responsible for neurofibrillary ‘tangle’ pathology in ad [10] whereby it undergoes conformational change, causing intraneuronal tangles which interrupt normal axonal messaging and ultimately cause cell death and atrophy [11, 12]. Recent data suggests that p-tau and amyloid may act synergistically to propagate toxic accumulation of both protein species [13, 14] (see Figure 1).

**Figure 2:**
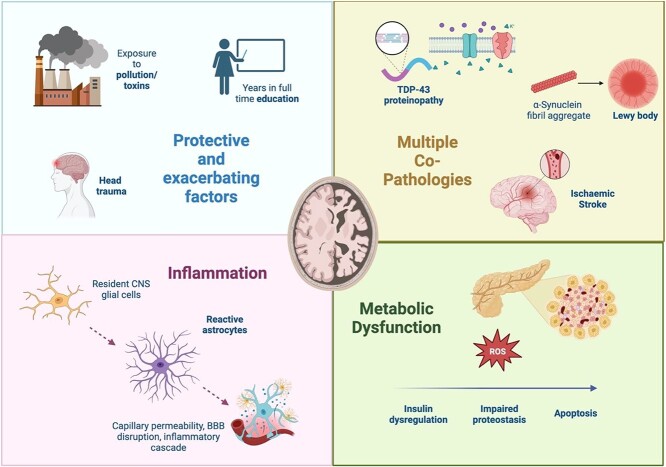
Additional pathophysiological processes known to influence Alzheimer’s Disease pathology in older adults. TDP-43: TAR (transactive response) DNA-binding protein 43. CNS: Central Nervous System. BBB: Blood–Brain Barrier. Image created with BioRender.com.

The degree to which amyloid plaques and tau tangles correlate with cognitive impairment in those with genetic predispositions is well established [15] and illustrated by persons with genetic APP or presenilin (PSEN1 or PSEN 2) mutations all directly causing toxic amyloid build-up and development of cognitive impairment [16]. However as brains age, there are often multiple factors causing neurodegeneration and cell death [17] with amyloid and tau pathology only two of multiple pathological causes of cognitive impairment [6]. The extent to which inflammation, metabolic dysfunction, vascular disease, dysfunctional proteostasis, alterations in lipid metabolism, cellular senescence and other neurodegenerative processes affect cognitive impairment in ad is a rapidly advancing field, beyond the scope of this review [18–20] (see Figure 2).

### Clinical presentation

The typical ad clinical phenotype is one of episodic memory loss, which may be noticed by the person themselves or by a suitable informant [21]. Atypical ad phenotypes include prominent initial visuo-spatial impairment (Posterior Cortical Atrophy [PCA]) or language impairment (logopenic-variant primary progressive aphasia). Rarely, individuals may present with behavioural or dysexecutive variants of ad [6]. Typically, ad may present as either MCI or established dementia, dependent on the functional status, as summarised below.

#### Mild cognitive impairment

MCI is a syndrome defined by objective cognitive changes without significant impairments in instrumental Activities of Daily Living (ADLs)—typically cognitive performance at least 1.5 standard deviations or greater below appropriate age and education-adjusted cut-off [22, 23]. Where due to ad, MCI typically presents with impairments in episodic memory that do not interfere with day-to-day function. In practice, MCI has an annual conversion rate to dementia of 5–20% [23, 24]; however, cognitive performance may deteriorate, remain stable, or even improve over time [25].

MCI can be sub-classified as amnestic or non-amnestic, and single or multiple domains affected [26]. Patients with amnestic MCI may have a conversion rate of up to 40% over 5 years [27]; however, those at greatest risk have multidomain amnestic MCI (impairment in episodic memory accompanied by another domain deficit—frequently visuospatial, executive or language impairment) [28, 29]. Conversion rates from MCI to dementia in individuals with MCI and positive biomarkers of ad pathology—discussed below—may be up to 11-fold higher compared to individuals with MCI and negative ad biomarkers [30].

#### Dementia

Dementia is a clinical syndrome where cognitive impairment results in impairments in ADLs and day-to-day function which may be further characterised as mild, moderate and severe [6]. ad is the most common pathology causing dementia in older adults; however, other conditions such as Dementia with Lewy Bodies (DLB), Fronto-Temporal Dementia (FTD), Limbic-predominant Age-related TDP-43 Encephalopathy (LATE) and cerebrovascular disease (causing cognitive impairment or dementia) give rise to co-pathology, which is encountered frequently in older adults [31].

### AD diagnostic criteria and biomarker-assisted definitions

**Figure 3:**
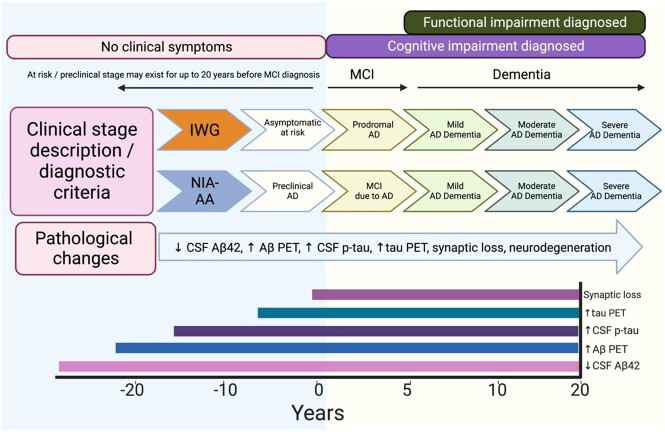
Diagnostic criteria and disease stages of ad over a chronological continuum. NIA-AA: National Institute of Aging and Alzheimer’s Association. CSF: Cerebro-spinal Fluid. Aβ42: amyloid-beta 42. Aβ-PET: amyloid-beta positron emission tomography. Image created with BioRender.com.

For several decades, the prevailing diagnostic criteria for ad (NINCDS-ADRDA) were based on clinical criteria alone and graded as mild/moderate/severe based on functional dependency [32]. It has since been estimated that up to one-third of those diagnosed based on these criteria have no evidence of underlying ad neuro-pathology when examined post-mortem [33]. The 2018 National Institute of Aging and Alzheimer’s Association (NIA-AA) research criteria advocate for a biological classification of ad in a clinical context based on available biomarkers [34] and initiated the diagnosis of ad based on ATN criteria (A: amyloid +/–, T: Tau +/−, N: neurodegeneration +/−). Recent criteria from the International Working Group (IWG) assert that biomarker status must only be interpreted in the presence of an appropriate clinical phenotype (‘phenotype-positive ad’) [6]. A further evolution to these criteria which is under review has been drafted and suggests that the presence of amyloid pathology alone may be sufficient to diagnose ad. These criteria are briefly summarised in Table 1 and Figure 3.

**Table 1:** Table of frameworks for diagnosing Alzheimer’s disease.

Frameworks for diagnosing AD		
Descriptor, year	Clinical	Biological
NINCDS-ADRDA (1984) [32]	Dementia—memory or cognitive changes plus another cognitive impairment	Nil
IWG Research Criteria (2007) [94]	Designed for research purposes – required diagnosis of ‘amnestic syndrome of hippocampal type’	CSF biomarkers, MRI atrophy, FDG- PET hypometabolism, amyloid PET positive, or ad autosomal dominant mutation
NIA-AA (2011) [95]	Clinical diagnosis of MCI (amnestic or non-amnestic) or dementia	Amyloid β marker (CSF or PET) or marker of degeneration (CSF tau, p-tau, FDG-PET, or structural MRI)
NIA-AA (2018) [34]	No clinical diagnosis necessary Introduced concept of ATN classification	Amyloid β marker (CSF or PET) and tau marker (CSF or PET)
IWG (2021) [6]	Must have clinical diagnosis of one of: Amnestic variant Posterior cortical atrophy Logopenic variant primary progressive aphasia Behavioural or dysexecutive frontal variant Corticobasal syndrome	Amyloid β marker (CSF or PET) and tau marker (CSF or PET)

NINCDS-ADRDA: National Institute of Neurological and Communicative Disorders and Stroke and the Alzheimer’s Disease and Related Disorders Association. IWG: International Working Group.

### Achieving a clinical–biological diagnosis of AD: advances and challenges

As mentioned, the diagnosis of ad based on clinical phenotype alone may not accurately reflect underlying pathological correlates. As a result, there have been substantial developments in the use of diagnostic biomarkers for ad pathology. The most commonly studied biomarkers include Cerebro-Spinal Fluid (CSF) and Positron Emission Tomography (PET) imaging which demonstrate changes in amyloid and p-tau seen with ad pathology. Blood-based biomarkers are an area of intense research interest and are likely to become clinically available in the coming years [35, 36].

#### Fluid biomarkers

Diagnostic Lumbar Puncture (LP) involves assessing CSF levels of Amyloid Beta (Aβ) and p-tau which may demonstrate pathological changes consistent with ad (depleted Aβ-42 and elevated p-tau) [6]. While LP is invasive and not without risk, they are typically well-tolerated as elective procedures in MAS [37].

In the UK National Audit in 2021, approximately 2% of individuals presenting to MAS had additional specialist investigations performed—one of which was diagnostic LP [38]. At present, the National Institute for Clinical Excellence (NICE) recommends diagnostic LP where the diagnosis is uncertain and ad is suspected [39]. A recent meta-analysis has suggested that diagnostic LP in a memory clinic setting significantly improves clinicians’ confidence in diagnosing ad and influences patient management [40]. A recent European Consensus group has been convened on the use of diagnostic biomarkers in MCI and early dementia, the results of which may give a more realistic reflection of current practice [41].

A specific concern in the use of diagnostic biomarkers in older adults with ad is the presence of co-pathology. In later life dementia, multiple proteinopathies are more frequent than single causes of neurodegeneration [6]. These include the presence of alpha-synuclein, TDP-43 proteinopathies (including LATE), non-ad tauopathies, vascular pathology and hippocampal sclerosis which may be present in over half of clinically defined ad [42, 43]. Many older adults also have evidence of ad pathology but no cognitive symptoms [44, 45]. When used in older adults, biomarkers should only be incorporated alongside an appropriate clinical presentation and interpreted by those with expertise in their use, preferably in the context of multi-disciplinary consensus MAS diagnostic meetings.

CSF analysis for ad biomarkers may be particularly useful in predicting conversion to dementia in older adults with MCI [46] with a sensitivity and specificity approaching 95% and 87%, respectively [47, 48]. However, it has been estimated that the risk of developing dementia with positive ad biomarkers depends on age, with a Hazard Ratio (HR) of developing ad dementia at age 65 of 21.4 and a HR of 4.9 age 85 for the same ad biomarker profile [7, 49]. Issues regarding lack of precise cut-offs in older adults presenting with MCI or early dementia require further longitudinal population-based studies and should be carefully resolved as the field moves forward [50].

Recent studies have suggested that more precise clinical–biological diagnosis of ad may result in reduced care costs, delayed institutionalisation and even reduced mortality results which warrant replication [51, 52]. Achieving a clinical–biological diagnosis of ad in older adults with MCI/early dementia may identify those suitable for novel Disease-Modifying Therapies (DMTs) should become available for older adults in the future.

#### Imaging

Basic structural neuroimaging should be performed where feasible, primarily to exclude occult central pathologies. There is marked regional and geographic variation in practice—in 2021, less than half of those assessed in MAS were referred for a structural neuroimaging in the National Dementia Audit in England and Wales [38]. The recently published Model of Care for Dementia in Ireland recommends neuroimaging for all new MAS diagnoses—preferably Magnetic Resonance Imaging (MRI) [53]. MRI hallmark findings in ad include identification of Medial Temporal lobe Atrophy (MTA), and specific patterns of cortical atrophy—especially in atypical presentations. MTA atrophy can be objectively graded from 0 to 4 [54] and predicts both progression to dementia from MCI and underlying ad neuropathology [55, 56]. Standardised scores are also available for the quantification of vascular burden (Fazekas score) and degree of posterior parietal atrophy (Koedam score) [57, 58].

Fluoro-DeoxyGlucose PET (FDG-PET) is useful for detailing hypometabolism in specific areas that are associated with ad (e.g. precuneus, medial temporal and posterior parietal areas), and these findings on FDG-PET can predict conversion to ad dementia in those with MCI [59, 60]. Amyloid PET imaging is becoming more widely available with three European licenced tracers available (18F-florbetaben, 18F-flutemetamol, 18F-florbetapir) and can be used as a non-invasive alternative to establishing amyloid burden [61]. Amyloid-PET and tau-PET imaging offer a non-invasive, but currently more expensive means of detecting amyloid plaque and tau pathological change consistent with ad.

### Achieving an accurate and timely diagnosis of AD in older adults

Despite these advances, ad remains underdiagnosed in older adults [62]. An accurate diagnosis may reduce carer strain, psychological morbidity and improve quality of life for patients with ad [63–65]. However, the perception that cognitive decline is part of normal ageing, the enduring stigma around a diagnosis of ad and the concept that there are few effective treatments available for ad persist and may mean that some older adults are reluctant to present with memory or cognitive symptoms. Internationally, there remain significant barriers in access to MAS especially in low- and middle-income countries [66].

### Diagnostic pathways in older adults with cognitive impairment

General practitioners are often the first clinician who recognise cognitive decline in an older adult and may refer on to a MAS. Increasingly, MAS services strive for multi-disciplinary and multi-modal assessment and achieve final diagnosis based on multi-disciplinary consensus. Currently, in the UK context, the National Audit of Dementia reports that there are approximately 222 MAS in England and 25 in Wales in 2021 [38]. In Ireland, there are currently 27 MAS units with the proposed model of care aiming to deliver five Regional Specialist Memory Centres (RSMC) nationally [53, 67]. Supplementary Figure 1 illustrates the core constituent members of a MAS/RSMC.

### Management of AD in older adults

Once a diagnosis of MCI or dementia due to ad is reached, it should be fully disclosed bearing in mind the patient’s wishes—only a minority of older adults would favour not being informed of the diagnosis [64]. At present, over-emphasis on pharmacological strategies should be avoided as ad, like other conditions in older adults, responds better to a multi-modal and multi-disciplinary care plan [64]. Clinicians should address potentially modifiable risk factors for further cognitive decline, informed by the principles of a brain health approach. It should be emphasised that while ad may cause functional impairment, morbidity and increase mortality, many older adults with ad lead fulfilling lives with good quality of life in comparison to their peers [68].

#### Targeted lifestyle and brain health interventions

In recent years, the concept of multifactorial lifestyle interventions to prevent ad and other causes of dementia has been established—given that up to 40% of the population-attributable risk for dementia is potentially due to modifiable risk factors [65]. In early ad (MCI or mild dementia due to ad), a comprehensive lifestyle intervention approach, guided by CGA, aims to mitigate the risk posed by remediable factors contributing to cognitive impairment. This includes correcting visual and hearing impairments, alcohol and smoking cessation, implementing a Mediterranean diet, assessment and modification of vascular risk factors, cessation of contributory medications (e.g. benzodiazepines and anticholinergics) and assessment and management of sleep, mood, anxiety, social isolation and loneliness. The landmark Finnish Geriatric Intervention Study to Prevent Cognitive Impairment and Disability (FINGER) study has demonstrated the potential of a multi-domain intervention inclusive of vascular risk factor monitoring, cognitive stimulation, exercise and diet modification to maintain or even improve cognitive function in older adults at risk of dementia [69].

**Figure 4:**
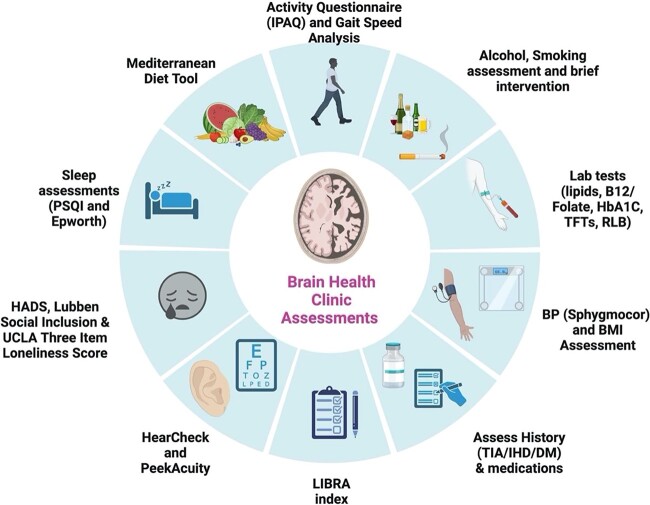
Brain Health Clinic assessments and recommended clinical assessment tools. IPAQ: International Physical Activity Questionnaire. TFT: Thyroid Function Test. RLB: Renal, Liver, Bone laboratory profile. BP: Blood Pressure. BMI: Body Mass Index. TIA: Transient Ischaemic Attack. IHD: Ischaemic Heart Disease. DM: Diabetes Mellitus. LIBRA: Lifestyle For Brain Health Index. HADS: Hospital Anxiety and Depression Scale. PSQI: Pittsburgh Sleep Quality Index. Image created with BioRender.com.

A recent Randomised Controlled Trial (RCT) of a peer-supported exercise intervention has demonstrated benefits in executive function, attention and working memory in older adults with MCI [70], adding to results of an earlier meta-analysis in MCI [71] and established dementia [72]. Structured exercise may also mitigate against sarcopenia, frailty and falls in older adults with early ad [73]. There is also strong RCT evidence that Cognitive Stimulation Therapy (CST) may be beneficial in mild to moderate dementia with group CST currently advised by NICE [39, 74]. Personalised nonpharmacological brain health approaches, vascular risk factor modification and exercise interventions should be offered to all older adults with ad-MCI or mild dementia due to ad, with CST offered to those with established dementia. Sample components of a Brain Health Clinic approach are illustrated in Figure 4.

#### Pharmacological management of dementia due to AD

Cholinesterase inhibitors have for decades been the mainstay of symptomatic treatment of dementia due to ad. Evidence from a comprehensive Cochrane review notes that after 26 weeks, treatment with cholinesterase inhibitors was associated with better cognitive function on both the MMSE of +1.05 and the ADAS-Cog of −2.67, improvements in ADLs and on the clinician-rated global assessment [75]. However, response is variable and due to side effects (gastrointestinal upset, fatigue and muscle cramps) they are not tolerated by around one-third of older adults [76]. In moderate–severe dementia due to ad, memantine (an NMDA receptor antagonist) is frequently used which exerts moderate benefits in combination with cholinesterase inhibitors—namely a small clinical benefit in clinical global rating (−0.21), slight improvements in ADL performance, behaviour and mood (−1.84 points on the Neuro-Psychiatric Inventory) but no impact on cognitive function [77]. NICE recommends cholinesterase inhibitors (donepezil, rivastigmine, galantamine) for mild–moderate ad, memantine for all stages of ad if intolerant of cholinesterase inhibitors and combination of memantine and cholinesterase inhibitors in severe ad [39]. Notably, secondary analysis of the DOMINO-ad trial noted that in moderate–severe ad, discontinuation of cholinesterase inhibitors may be associated with functional decline [78].

#### Emerging treatments for older adults with AD

Recent phase 3 RCTs have demonstrated potential efficacy of immunotherapies targeting aggregated forms of Aβ (aducanumab, lecanemab and donanemab) in slowing clinical progression of early ad (MCI due to ad and mild dementia due to ad) defined by clinical–biological diagnosis with positive ad biomarkers. Typically, the patients included in these RCTs are younger, more educated, with fewer medical comorbidities and less frail than real-world patients. Notably, based on Appropriate Use Recommendations (AURs) for aducanumab and lecanemab, a significant number of older adults presenting to MAS would be eligible for treatment with these agents, should they become available [79–82].

In the recent CLARITY-ad study, lecanemab demonstrated a statistically significant effect on the Clinical Dementia Rating Sum-of-Boxes (CDR-Sb) at 18 months (a change of −0.45) in older adults with MCI and early dementia due to ad [83]. More recently, in TRAILBLAZER-ALZ 2, a phase 3 RCT of donanemab in MCI and early dementia due to ad, treatment resulted in a slowing of clinical progression on the CDR-Sb over 76 weeks (a change of −0.7) [84].

One of the greatest concerns arising from the use of immunotherapies against Aβ centres around the development of Amyloid-Related Imaging Abnormalities (ARIAs). These imaging abnormalities, divided into oedema (ARIA-E) or haemorrhage (ARIA-H), were observed in 36.8% of those on donanemab vs 14.9% on placebo, and up to 21% of those on lecanemab vs 9.5% on placebo. The majority of ARIA were asymptomatic, mild–moderate in nature and resolved over the course of a number of months; however, in a proportion of patients, they were severe and can be fatal. ARIA require an increase in the frequency of MRI monitoring to ensure resolution and may require hospital admission to manage in severe cases. Further concerns of novel DMTs centre around the increased treatment and monitoring burden, with regular intravenous infusions (required every 2 weeks in the case of lecanemab) and regular MRI scans to monitor for ARIA balanced against potential clinical benefit. Another potential concern is regarding the side effect of brain volume loss, which is apparent across all anti-amyloid therapies, may measure up to 5 ml of atrophy over 12–18 months [85] and is of unclear clinical significance [86]. Whether these medications prove clinically beneficial and cost-effective in real-world patients outside of the phase 3 clinical RCTs will become apparent in time, with real-world monitoring through registries such as the Alzheimer’s Network for Treatment and Diagnostics (ALZ-NET).

### Implications of AD diagnostic and treatment advances in older adults

In considering the above potential advances in ad, it is noteworthy that patients and families value an accurate clinical–biological diagnosis, with many patients wishing to undergo invasive tests such as LP to achieve this [87]. While recent trial results have demonstrated statistically significant effects, the real-world clinical significance of these is not currently clear and there is an urgent need to evaluate clinical trial outcomes and how they translate into real world benefit for older adults with ad [88, 89]. Many of the observed effect sizes of DMTs on the primary outcome measure in phase 3 RCTs in ad—namely the CDR-Sb—are less than the published minimal clinically important differences in ad [90]. Moving forward, ad trials should consider the impact of any potential DMT on patient-centred and patient-reported outcomes including quality-of-life, effects on institutionalisation, burdensome aspects of ad care and the development of other geriatric syndromes. The use of ad DMTs, for which over 30,000 individuals in the UK could be eligible [91], would require a paradigmatic shift in existing diagnostic and treatment pathways. The includes biomarker-assisted diagnostics, neuroimaging, access to infusion suites and workforce training to deliver and monitor DMTs.

The potential annual cost of delivery of DMTs in the UK could be up to £9 billion [92] and in the EU states could exceed €133 billion [93]. It is our view that in this context, we as geriatricians must advocate not only for increased access to enhanced diagnostic and treatment pathways for older adults with ad, but urge health systems to enhance access to multidisciplinary, holistic and gerontologically-informed health and social care for all older adults with ad. This is especially true for those with severe ad or those ineligible for potential new DMTs. New advances in ad diagnosis and treatment must not divert existing funding from health and social care services for older adults with ad.

## Conclusion

A timely, accurate diagnosis of ad with access to appropriate multidisciplinary support and treatment improves quality of life and reduces carer strain. Biomarkers have the potential to help support this process but should be used as part of a clinical-biological diagnosis by specialists with experience in interpreting the results. Co-pathology is frequent in older adults, and the recent advances in ad biomarker-assisted diagnosis should not be to the detriment of the diagnosis and treatment of other causes of cognitive impairment. The development of new immunological therapies for ad is exciting for patients and professionals alike but the results of current trials should be considered cautiously and their findings’ applicability to more typical patient groups need to be weighed carefully. We believe that a patient-centred-approach using the tenets of CGA remains a robust framework into which new diagnostic tools and treatments can be adopted.

## Supplementary Material

aa-23-1918-File002_afae005
