# Supplementary material for: New horizons in the diagnosis and management of Alzheimer’s Disease in older adults
Source: Age Ageing. 2024 Feb 10;53(2):afae005. doi: 10.1093/ageing/afae005 (PMC10859247; doi:10.1093/ageing/afae005)
Supplement: aa-23-1918-File002_afae005 [file aa-23-1918-file002_afae005.docx]

**New Horizons in the Diagnosis and Management of Alzheimer Disease in Older Adults**

**Supplementary Figure 1. Constituent Members of a Memory Assessment Service**

**
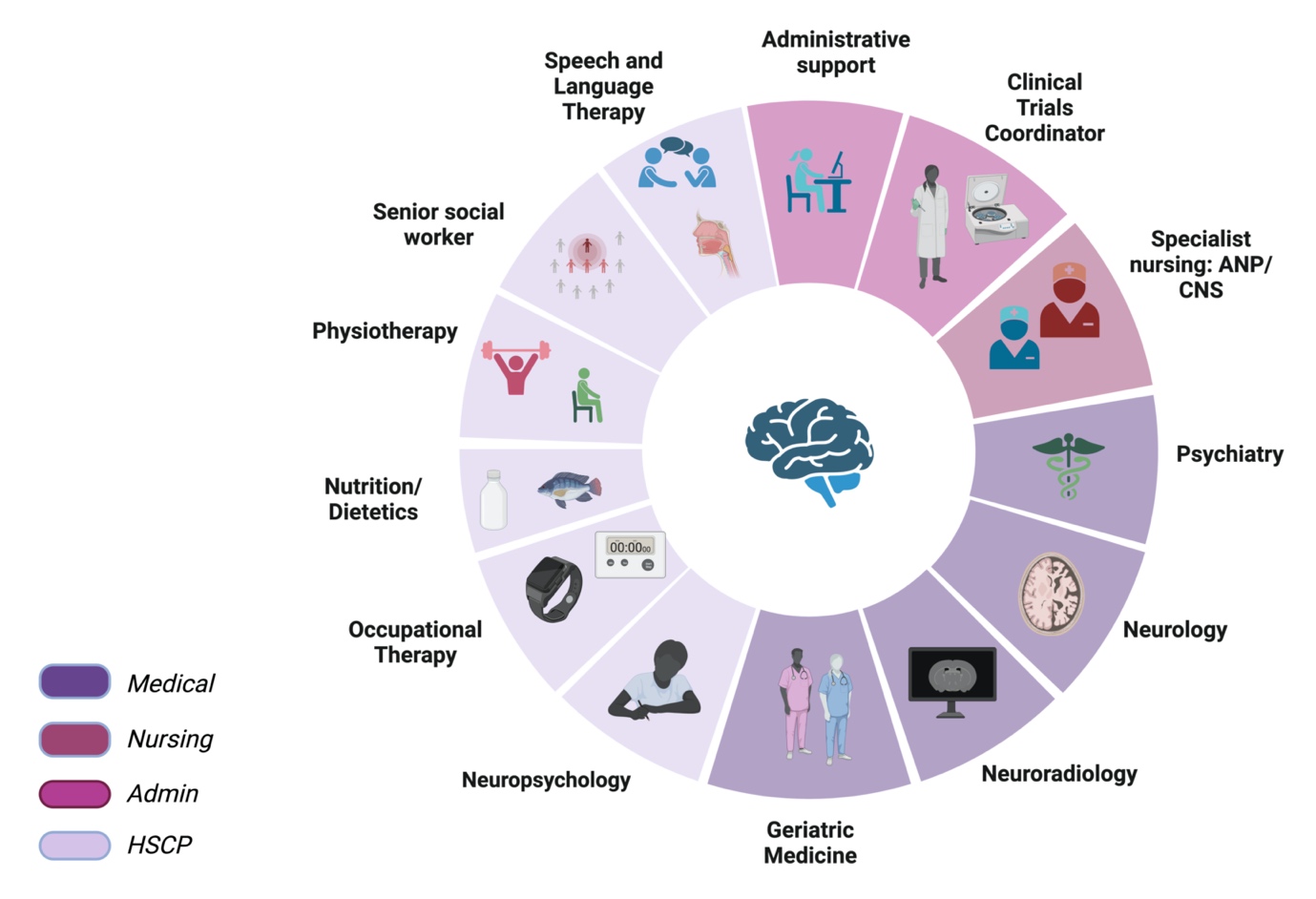
**

HSCP: Health and Social Care Professional. ANP: Advanced Nurse Practitioner. CNS: Clinical Nurse Specialist
